# Recombination in Avian Gamma-Coronavirus Infectious Bronchitis Virus

**DOI:** 10.3390/v3091777

**Published:** 2011-09-23

**Authors:** Sharmi W. Thor, Deborah A. Hilt, Jessica C. Kissinger, Andrew H. Paterson, Mark W. Jackwood

**Affiliations:** 1 Department of Population Health, Poultry Diagnostic and Research Center, College of Veterinary Medicine, University of Georgia, Athens, GA 30602, USA; E-Mails: sthor@uga.edu (S.W.T.); dhilt@uga.edu (D.A.H.); 2 Department of Genetics, Center for Tropical and Emerging Global Diseases, University of Georgia, Athens, GA 30602, USA; E-Mail: jkissing@uga.edu; 3 Plant Genome Mapping Laboratory, Departments of Crop and Soil Sciences, Plant Biology and Genetics, University of Georgia, Athens, GA 30602, USA; E-Mail: paterson@plantbio.uga.edu

**Keywords:** gamma coronavirus, avian coronavirus, infectious bronchitis virus, genome, recombination

## Abstract

Recombination in the family *Coronaviridae* has been well documented and is thought to be a contributing factor in the emergence and evolution of different coronaviral genotypes as well as different species of coronavirus. However, there are limited data available on the frequency and extent of recombination in coronaviruses in nature and particularly for the avian gamma-coronaviruses where only recently the emergence of a turkey coronavirus has been attributed solely to recombination. In this study, the full-length genomes of eight avian gamma-coronavirus infectious bronchitis virus (IBV) isolates were sequenced and along with other full-length IBV genomes available from GenBank were analyzed for recombination. Evidence of recombination was found in every sequence analyzed and was distributed throughout the entire genome. Areas that have the highest occurrence of recombination are located in regions of the genome that code for nonstructural proteins 2, 3 and 16, and the structural spike glycoprotein. The extent of the recombination observed, suggests that this may be one of the principal mechanisms for generating genetic and antigenic diversity within IBV. These data indicate that reticulate evolutionary change due to recombination in IBV, likely plays a major role in the origin and adaptation of the virus leading to new genetic types and strains of the virus.

## 1. Introduction

Avian infectious bronchitis virus (IBV) is a gamma-coronavirus in the family *Coronaviridae*, the order *Nidovirales*, and the genus *Coronavirus* that causes a highly contagious upper-respiratory disease of domestic chickens. In layer type birds it can cause a drop in egg production and some strains are nephropathogenic. Infectious bronchitis remains one of the most widely reported respiratory diseases of chickens worldwide despite the routine usage of attenuated live vaccines to control the disease. Control of IBV is difficult because there is little to no cross-protection between the numerous different serotypes of the virus.

Infectious bronchitis virus is an enveloped, single-stranded, positive-sense RNA virus with a genome length of approximately 27 kb. The 3′ end of the genome encodes four structural proteins; spike (S), envelope (E), membrane (M) and nucleocapsid (N) as well as several non-structural proteins [1]. The S glycoprotein of IBV forms projections on the surface of the virion. Spike is post-translationally cleaved into S1 and S2 subunits with the S1 subunit forming the outermost portion and S2 forming a stalk-like structure that is embedded in the viral membrane. The S1 subunit contains hypervariable regions that play a role in attachment to host cell receptors, and it contains conformationally-dependent virus-neutralizing and serotype-specific epitopes [2,3]. Spike is also involved in membrane fusion and viral entry into the host cell. The E and M proteins are integral membrane proteins involved in assembly of the virus. The N protein is closely associated with the viral genome and plays a role in replication. The 5′ two-thirds of the genome, approximately 21 kb, encodes two polyproteins 1a and 1ab. A minus one frame-shift mechanism is used to translate the 1ab polyprotein. The polyproteins are post-translationally cleaved into 15 non-structural proteins (nsps), nsp 2–16 (IBV does not have an nsp1) that make up the replication complex. Key nsps encoded, include a papain-like protease 2 (PLP2) within nsp 3, a main protease (Mpro) within nsp 5, and the RNA-dependent RNA-polymerase (RdRp) within nsps 11 and 12.

Genetic diversity in coronaviruses is due to adaptive evolution driven by high mutation rates and genetic recombination [4]. High mutation rates are attributed to minimal proof reading capabilities associated with the RdRp. Recombination is thought to be due to a unique template switching “copy-choice” mechanism during RNA replication [5]. Evidence of recombination among strains of IBV has been observed both experimentally and in the field [6–11]. The emergence of several alpha- and beta-coronaviruses has been attributed to recombination [12,13] but only recently was recombination shown to be the mechanism behind the emergence of a novel gamma-coronavirus, turkey coronavirus (TCoV) [14]. Although “hot spots” of recombination in the genome of IBV have been reported [9,15], a thorough study of recombination using multiple different strains across the entire genome has not been conducted.

In this study we sequenced and analyzed the entire genome of eight IBV strains that represent different serotypes that have not been previously sequenced, and we compared these sequences with other gamma-coronavirus full-length genome sequences available in GenBank for evidence of recombination [16]. Different serotypes of field viruses and vaccine type viruses were selected to provide a wide variety of sequences potentially capable of contributing gene fragments to recombinants.

## 2. Results and Discussion

### 2.1. Sequence Analysis

The full-length genomes of eight isolates of IBV were sequenced at 5× to 10× coverage, and the consensus sequences were assembled. The genome size (see the end of the 3′UTR in Table 1), organization of the genome, and the location and size of the open reading frames (ORFs) are listed in Table 1 for each of the viruses. The gene order is the same for all the viruses examined; 5′UTR-1a/ab-spike-3a-3b-Envelope-Membrane-4b-4c-5a-5b-Nucleocapsid-3′UTR. In addition, the genomes for CAV/CAV56b/91, DE/DE072/92, FL/FL18288/71, Mass/H120, Iowa/Iowa97/56 and JMK/JMK/64 contain ORF 6b between nucleocapsid and the 3′UTR.

**Figure 1.**
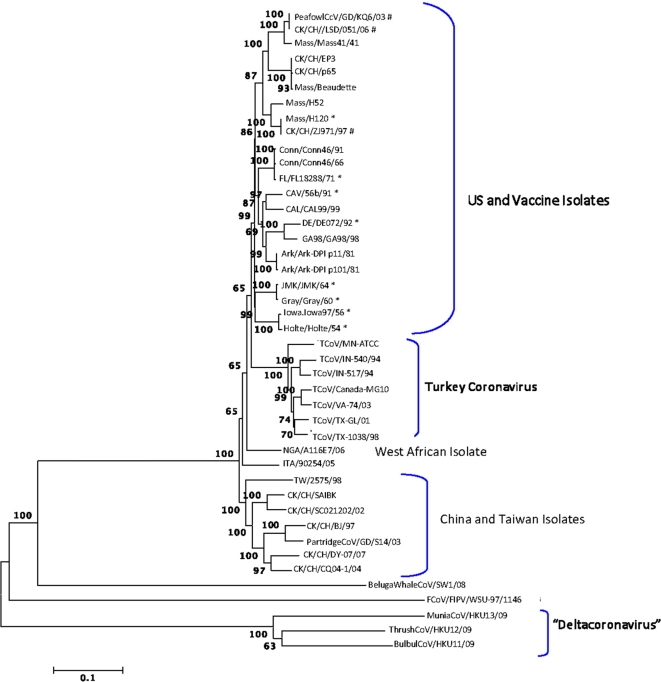
Neighbor-joining method used to infer evolutionary history using full genomic sequence data available for the gamma-coronaviruses. The p rcentage of replicate trees in which the associated taxa clustered together in a bootstrap test of 1000 replicates is shown next to the branches. The p-distance scale is presented at the bottom of the figure. An asterisk (*) indicates a strain newly sequenced in this study. A number sign (#) indicates strains isolated in China that grouped with vaccine strains of infectious bronchitis virus (IBV).

The full-length genomes were aligned and phylogenetic trees were constructed using the Neighbor-joining, Minimum Evolution, Maximum Parsimony and UPGMA programs in MEGA4 [17]. The trees all had similar topology and bootstrap support, and a representative tree is shown in Figure 1. The feline coronavirus FCoV/FIPV/WSU-79-1146 and the beluga whale virus BelugaWhaleCoV/SW1/08 were included as out-groups. The wild bird viruses isolated from a munia (MuniaCoV/HKUY13/09), thrush (ThrushCoV/HKU12/09) and bulbul (BulBulCoV/HKU11/09) formed a unique clade, which is not surprising as this group might represent a new coronavirus genus provisionally designated Deltacoronavirus [18]. The remaining viruses separated into clades consisting of IBV isolates from the US and vaccine viruses, TCoV isolates, an IBV isolate from West Africa and IBV isolates from China and Taiwan.

Vaccines for IBV used in commercial poultry include the serotypes Mass, Conn, DE and Ark. The PeafowlCcV/GD/KQ6/03, CK/CH/LSD/051/06 and CK/CH/ZJ971/97 strains from China grouped with Mass type viruses indicating that they are closely related, which is not surprising since Mass type vaccines are used in China. The overall percent similarities between the various strains are listed in Supplemental Table 1. All IBV genomes examined are greater than 80% similar at the nucleotide level.

### 2.2. Recombination Analysis

**Figure 2.**
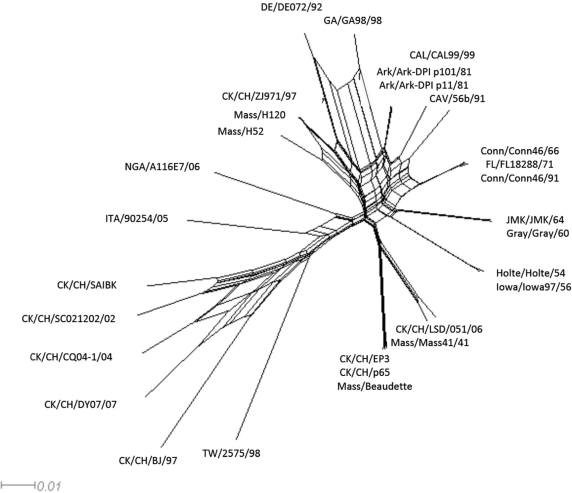
Neighbor-net for the avian gamma-coronavirus IBV. The networked relationships are shown to indicate the presence of reticulate events. Boxes imply the likelihood of recombination.

Recombination among coronaviruses reduces mutat onal load, creates genetic variation, and can result in the emergence of new strains [19]. However, evolutionary history is **t**raditionally represented using a strictly bifurcating phylogenetic tree, which implies that nce two lineages are created they subsequently never interact with each other. When evolutionary events such as reassortment, horizontal gene transfer or recombination occur, reticulations among the phylogenetic tree branches can result. Using the Neighbor-net analysis we observed evidence of networked relationships (represented by boxes, in Figure 2) among **t**he analyzed sequences. Since the boxes only imply the possibility of recombination, we conducted a pairwise homoplasy index (PHI) test, which showed a significant difference in the compatibility between closely linked sites (p < 0.0001) supporting the occurrences of recombination among the viruses [20].

The Recombination Detection Program 4 (RDP4) [21,22] w s used to identify recombination breakpoint positions in full-length IBV genome sequences and the data for 50 of a total 135 unique transferred fragments with statistical support of p ≤ 1 × 10^−12^ are listed in Table 2. The full-length genomes available in the database for CK/CH/EP3, CK/CH/p65, and Mass/Beaudette were excluded from the analysis because they are viruses not found in the field. The recombination programs can be used to detect recombination without reference sequences, and our analysis was conducted without regard to date of isolation because that information was not available for some of the viruses. Although the programs attempt to identify major and minor parent sequences contributing to each recombinant, the data reported herein only represents sequences in other viruses that are most closely related to the sequence surrounding the transferred fragment (major sequence) and the sequence closely related to the transferred fragment (minor sequences) and doesn’t imply origin or source of the transferred fragment. In many cases, the transferred fragment has undergone mutations making it difficult to identify all the endpoints for the major and minor sequences. In addition, some of the transferred fragments overlap suggesting that recombinations have occurred between recombinant viruses.

Twenty-five IBV strains were examined and the viruses with the most transferred fragments in Table 2 are CAV/56b/91 and Mass/H52 both with 8 fragments, and CK/CH/LSD/051/06 and GA98/0470/98 both with 7 fragments. The strains with the fewest transferred fragments are Iowa/Iowa97/56 and TW/2575/98 with only 2 transferred fragments and the CK/CH/BJ/97, Holte/Holte/54, and NGA/A116E7/06 strains with only 1 transferred fragment. The Ark/Ark-DPI-p11/81 and Ark/Ark-DPI-p101/81 strains are the same virus that was passaged 11 and 101 times in embryonated eggs, respectively. Both viruses share identical transferred fragments indicating that they have identical recombination history. In addition, Conn/Conn46/66 and Conn/Conn46/91 share the same recombination history (4 identical transferred fragments). The Conn/Conn46/66 field virus was used to produce an attenuated live vaccine, which is currently used in commercial poultry. Viruses that share the same recombination history are likely derived from the same parent virus suggesting that Conn/Conn46/91 is reisolated Conn vaccine derived from the Conn/Conn46/66 virus. The FL/FL18288/71 virus also shares all 4 transferred fragments with the Conn viruses, however; FL/FL18288/71 and Conn viruses are different serotypes suggesting that FL/FL18288/71 is a field virus that emerged due to point mutations accumulating in spike over time rather than from recombination.

All 6 of the transferred fragments in CK/CH/ZJ971/97 are identical to all 6 of the transferred fragments in vaccine strain Mass/H120, providing compelling evidence that CK/CH/ZJ971/97 is reisolated Mass/H120 vaccine. That observation was also reported by Zhang *et al.* [23]. It is interesting that Mass/H52 (8 transferred fragments) and Mass/H120 (6 transferred fragments) share only 5 identical transferred fragments. The Mass/H52 and Mass/H120 viruses were isolated *circa* 1955 in the Netherlands and it is widely accepted that H stands for Holland, but it actually stands for Houben, the owner of the broiler farm where the viruses were isolated [24]. It is thought that Mass/H120 was derived from Mass/H52 but the actual relationship between the viruses is not certain. Our data indicates that they are not necessarily parent and progeny but they are closely related.

The Gray/Gray/60 and JMK/JMK/64 viruses share 99.7% nucleotide similarity across the entire genome and have 4 identical transferred fragments with JMK/JMK/64 having one additional fragment located in the 5′UTR, which is not found in Gray/Gray/60. It is well known that the Gray/Gray/60 virus is nephropathogenic, whereas the JMK/JMK/64 virus is strictly respirotropic. Perhaps sequence differences in the 5′UTR, which is involved in replication of the viral genome, play a role in the different pathobiologies observed for these viruses.

There is evidence that some transferred fragments in field viruses come from vaccines. As an example, CK/CH/LSD/051/06 has 3 of 7 and 2 of 7 transferred fragments in common with vaccine strains Mass/H52 and Mass/H120, respectively. In addition, the only fragments that USA viruses have in common with the viruses from China and Taiwan are fragments also associated with Mass type vaccines, which are used in both regions, providing further evidence that some of the fragments in field viruses come from vaccines. That result and the observation in Figure 1 that the viruses separated into clades based on geographic location also supports the conclusion that USA viruses have not recombined with Asian viruses.

A difference in the order of taxa in phylogenetic trees constructed from different regions of the genome is further evidence of recombination [25]. The ordering of taxa in sequential trees [26,27] was conducted and inconsistent phylogenetic relationships were observed for all of the examined virus strains across the entire genome, indicating a substantial amount of recombination (data not shown). There is a high number of breakpoints in the 1a region of the genome and immediately upstream of the S gene, which has been previously shown to be a ‘hot spot’ for recombination [9]. A phylogenetic compatibility matrix constructed at the 70% bootstrap level for 250 bp sequence fragments at 100 bp intervals also showed that recombination breakpoints were distributed throughout the IBV genomes (data not shown).

**Figure 3.**
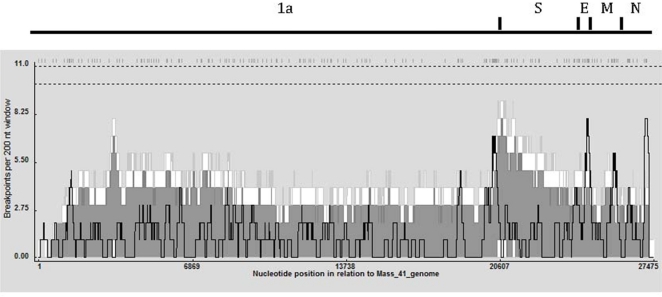
Recombination breakpoint distribution plot generated for IBV using the Recombination Detection Program 4 (RDP4) showing the detectable recombination breakpoints. The plot was constructed using a 200 bp window moved 1 nucleotide a a time along the length of the genome. Recombination breakpoint positions are shown as hash marks at the top of the figure. The dashed lines under the breakpoint positions represent 99% (upper) and 95% (lower) confidence thresholds for globally significant breakpoint clusters (defined as windows with more breakpoint positions than the maximum found in >95% of the permuted plots). The dark gray and white areas are 95% confidence and 99% confidence intervals, respectively, for local breakpoint clusters (defined as windows with more breakpoint positions than the maximum found in >99% of the windows at that location in permuted plots), and the black line indicates the breakpoint count within the 200 bp p window.

To determine recombination hot and cold spots, a recombination breakpoint distribution plot (Figure 3) was generated in RDP4 using a 200 nt window and 1,000 permutations [21]. No global hot-spot regions were observed in the 95% and 99% confidence thresholds (dotted lines at the top of the graph). The detectable recombination breakpoint positions are shown at the top of the figure and were distributed throughout the genome with a relatively high number clustered just upstream of the S gene. That region also had the highest breakpoint count within the 99% local hot/cold-spot confidence interval. A high number of breakpoints were also observed in the 1a region of the genome; nsp 2, nsp 3, and nsp 16, in the envelope and matrix protein genes and in a small area near the 3′UTR. Table 3 shows that nsp2, nsp3, nsp16 and spike genes were associated with the greatest number of transferred fragments, which is consistent with the location and number of breakpoints in Figure 3.

Recombination in the 1ab ORF area, which encodes the nonstructural proteins involved in the viral replication complex, has the potential to alter the pathogenicity of the virus [28]. The nsp 2 contains hydrophobic residues that likely anchor the replication complex to the Golgi [29]. The nsp 3 encodes the protease PLP2 which cleaves nsps 2, 3, and 4 and an area with ADP-ribose 1′-phosphatase (ADRP) activity. The protease PLP2 has been shown to have deubiquinating-like activity [30] and also to be a type I interferon (IFN) antagonist [31]. Changes in the amino acid composition of this area could affect the ability of the virus to replicate in a variety of cell types. The ADRP region of nsp 3 is conserved among coronaviruses [32,33], and a recent study suggested a biological role for the coronavirus ADRP in modulating the expression of pro-inflammatory immune modulators such as tumor necrosis factor alpha and interleukin-6 [34]. Recombination in this area could alter the pathogenicity of the virus by modulating host cytokine expression. The nsp16 is reported to be an S-adenosyl-L-methionine (AdoMet)-dependent RNA (nucleoside-2′*O*)-methyltransferase (2′*O*-MTase) responsible for capping the viral mRNA nascent transcripts [32]. An alteration in the efficiency of this protein could profoundly decrease not only viral replication but also pathogenicity. The spike glycoprotein of IBV on the surface of the virus plays a role in attachment to host cell receptors, membrane fusion and entry into the host cell. It also contains conformationally-dependent epitopes that induce virus-neutralizing and serotype specific antibodies [2,3]. We and others [6–8,10] have observed a relatively high number of breakpoints in and immediately upstream of spike, and changes to this region of the genome can result in the emergence of new genotypes and serotypes of IBV as well as new avian coronaviruses (*i.e*., TCoV). The envelope and matrix proteins are as ociated with virus assembly, and changes in those proteins could reduce the efficiency of virus particle formation and subsequent transmission of the virus. The 3′UTR is involved with binding of the viral RdRp and viral genome replication. Changes to the 3′UTR could affect replication efficiency and thus virulence of the virus.

## 3. Conclusions

In this study, evidence was obtained that recombination is occurring among avian coronavirus IBV isolates across their entire genome. Every sequence included in the analysis was recognized as a potential recipient of horizontally acquired sequences at some point in its viral evolutionary past. The nsp2, nsp3, nsp16 were associated with the greatest number of transferred fragments. In addition, the area immediately upstream of the spike gene had the highest number of recombination breakpoints. Breakpoints in the 1ab polyprotein gene have the potential to alter pathogenicity of the virus, and breakpoints near or in spike have the potential to lead to the emergence of new serotypes of IBV or new coronaviruses. Although the spike region determines the serotype of the virus, the remainder of the genome may be a mosaic of sequence fragments from a variety of gamma-coronaviruses. The only evidence of a gamma-coronavirus possibly recombining with an alpha or beta-coronavirus was the discovery of the mosaic nature of the SARS-coronavirus genome [35]. Although this type of recombination is possible it appears to be rare in nature.

In this study, we characterized recombination in the full-length genomes of avian gamma-coronavirus IBV strains from around the world. Our bioinformatic analysis was similar to a previous study on enteroviruses [36] and we found that recombination in IBV is more extensive than formerly thought, involving regions across the entire genome. Our data suggests that reticulate evolution due to a high frequency of recombination in IBV, likely plays a major role in the generation of new serotypes of the virus. The characterization, distribution and frequency of recombination breakpoints are important information that will further our understanding of the mechanisms behind the diversity and evolution of these viruses so that better control methods can be developed.

## 4. Materials and Methods

### 4.1. Viruses and Viral RNA Extraction

All of the viruses sequenced in this study (Table 4), were propagated in 9–11 day-old specific-pathogen-free (SPF) embryonated eggs as described [37]. Total RNA was isolated from 200 μL of allantoic fluid collected from the infected eggs using the High Pure RNA Isolation Kit (Roche Applied Science, Mannheim, Germany) following the manufacturer’s instructions.

### 4.2. RT-PCR Amplification and Sequencing

The amplification reactions were carried out using strand displacement RT-PCR or one step RT-PCR. Strand displacement RT-PCR uses a random (at the 3′ end) primer and an amplification primer. The sequence of the random primer was (AGCGGGGGTTGTCGAATGTTTGANNNN) and the sequence of the amplification primer was (AGCGGGGGTTGTCGAATGTTTGA). The RT-PCR reaction was carried out using the TaKaRa RNA LA PCR kit (Takara Bio. Inc., Otsu, Shiga, Japan) according to the manufacturer’s protocol. A DNA Engine Peltier thermocycler (Bio-Rad Laboratories Inc., Hercules, CA, USA) was used for the RT reaction, which included an RNA denaturing step at 65 °C for 10 min followed by 30 °C for 10 min, 42 °C for 60 min, 99 °C for 5 min, and 5 °C for 5 min. The PCR reaction was run on the same machine as the RT step and included a one-time initial denaturation step of 94 °C for 2 min, followed by 30 cycles of 94 °C for 30 s, 60 °C for 30 s and 72 °C for 3 min.

The PCR products were agarose gel purified using the QIAquick gel extraction kit (Qiagen, Valencia, CA, USA) according to the manufacturer’s protocol. The PCR products were cloned into the TOPOXL vector using the TOPOXL cloning kit (Invitrogen, Carlsbad, CA, USA) according to manufacturer’s protocol to prepare cDNA libraries for sequencing.

Plasmid DNA from the libraries of the cloned cDNA fragments for each virus was isolated using an alkaline lysis method modified for the 96-well format and incorporating both Hydra and Tomtek robots. Sequencing reactions were performed using the BigDye™ Terminator® Cycle Sequencing Kit Version 3.1 (Applied Biosystems, Foster City, CA, USA) and MJ Research (Watertown, MA, USA) thermocyclers. Sephadex filter plates were used to filter each reaction into Perkin-Elmer MicroAmp Optical 96-well plates. A 1/12-strength sequencing reaction on an ABI 3730 was used to sequence each clone from both the 5′ and 3′ ends.

Primers for one-step RT-PCR were specifically designed for each virus (Supplemental Table 2). Viral RNA was amplified using the Titan One Tube RT-PCR kit (Roche Diagnostics, Indianapolis, IN, USA) following manufacturer’s instructions. A DNA Engine Peltier Thermocycler (Bio-Rad Laboratories, Inc., Hercules, CA, USA) was used for the RT-PCR reaction, which had the following steps: one cycle of 42 °C for 60 min and 95 °C for 5 min, followed by 10 cycles of 94 °C for 30 s, 50 °C for 30 s, and 68 °C for 1 min 30 s, and then 25 cycles of 94 °C for 30 s, 50 °C for 30 s, 68 °C for 1 min and 30 s adding 5 s with each cycle.

The resulting PCR products were agarose gel purified using the QIAquick gel extraction kit (Qiagen, Valencia, CA, USA) according to the manufacturer’s protocol. The resulting cDNA was sequenced using ABI Prism BigDye Terminator Cycle Sequencing Ready Reaction Kit (Applied Biosystems, Foster City, CA, USA) following the manufacturer’s protocol. The reactions were prepared for sequencing by centrifugation through either a Centri-Sep column (Applied Biosystems, Foster City, CA, USA) or using the Edge system (EdgeBio, Gaithersburg, MD, USA) plate. The samples were sequenced at the Georgia Genomics Facility (University of Georgia, Athens, GA, USA).

### 4.3. Genome Assembly and Analysis

Chromatogram files and trace data were read and assembled using SeqMan Pro, and genome annotation was conducted with SeqBuilder (DNASTAR, Inc., Madison, WI, USA). Each sequence was aligned to a representative genome; Mass/Mass41/41 (GenBank accession #AY851295), or CAL99/CAL99/99 (GenBank accession #AY514485) as a backbone for genome assembly.

Whole genome analyses were generated and phylogenetic trees constructed with the Neighbor-Joining method with 1000 bootstrap replicates as well as with Minimum Evolution, Maximum Parsimony and UPGMA methods [17].

### 4.4. GenBank Accession Numbers

Virus genome sequences generated in this study were submitted to GenBank and assigned the following accession numbers: CAV/CAV56b/91 (GU393331), DE/DE072/92 (GU393332), FL/FL18288/71 (GU393333), Gray/Gray/60 (GU393334), Mass/H120 (GU393335), Holte/Holte/54 (GU393336), Iowa/Iowa97/56 (GU393337), JMK/JMK/64 (GU393338).

GenBank accession numbers for full-length sequences used as reference in this study are: Mass/Mass41/41 (AY851295), Mass/H52 (EU817497), Ark/Ark-DPI-p11/81 (EU418976), Ark-DPI-p101/91 (EU418975), CAV/CAV99/99 (AY514485), CK/CH/EP3 (DQ001338), CK/CH/p65 (DQ001339), Mass/Beaudette (NC_001451), NGA/A116E7/06 (FN430415), ITA/90254/05 (FN430414), TW/TW2575/98 (DQ646405), CK/CH/SC021202/02 (EU714029), CK/CH/ZJ971/97 (EU714028), CK/CH/BJ/97 (AY319651), CK/CH/SAIBK (DQ288927), CK/CH/LSD/051/06 (EU637854), CK/CH/DY07/07 (HM245923), CK/CH/CQ04-1/04 (HM245924), GA98/GA98/98 (GQ504723), PeafowlCcV/GD/KQ6/03 (AY641576), PartridgeCoV/GD/S14/03 (AY646283), TCoV/IN-540/94 (EU022525), TCoV/MN-ATCC (EU22526), TCoV/VA-74/03 (GQ427173), TCoV/TX-GL/01 (GQ427174), TCoV/IN-517/94 (GQ427175), TCoV/TX-1038/98 (GQ427176), TCoV/Canada-MG10 (EU095850) BulbulCoV/HKU11/09 (FJ376619), ThrushCoV/HKU12/09 (FJ376621), MuniaCoV/HKU13/09 (FJ376622), BelugaWhaleCoV/SW1/08 (NC_010646), FCoV/FIPV/WSU-79/1146 (DQ010921).

### 4.5. Detection of Networked Relationships and Recombination Break Points

We used Neighbor-net analysis to examine the IBV genomes for evidence of networked relationships and the pairwise homoplasy index (PHI) in SplitsTree (Version 4, Simmonics, University of Warwick, Coventry, UK) [20,38,39] to statistically determine the likelihood of recombination. In addition, the IBV genomes were examined for recombination breakpoints using the Recombination Detection Program (RDP4, Version 4, Simmonics, University of Warwick, Coventry, UK) [21,22]. Unless otherwise stated, default settings were used in all of the programs. The specific algorithms used were RDP [40], GENECONV [41], BOOTSCAN/RESCAN [40], MAXIMUM CHI SQUARE [42], CHIMAERA [43], SISCAN [44], and 3Seq [45]. We used more than one method to analyze the data because evaluation of these recombination detection methods using both simulated and empirical data showed that the results from only a single method were not very reliable [46]. Automasking was used for optimal recombination detection. The RDP analysis was run without a reference and a window size of 60, BOOTSCAN window size was increased to 500, MAXCHI and CHIMAERA number of variable sites per window was increased to 120, and the window size and step size for SISCAN was increased to 500 and 20, respectively. The window sizes were increased from their default settings because IBV has a high mutation rate, which can mask recombination signals. Increasing the window size was shown to increase the ratio of recombination signals relative to mutational “noise” [47].

### 4.6. Phylogenic Analysis of Sequential Genome Fragments

Inconsistent phylogenetic relationships between different regions of the viral genome provide further evidence of genetic recombination. Herein, we examined the order of avian gamma-coronavirus IBV strains in phylogenetic trees generated from sequential genome fragments using TreeOrder Scan (Version 1.6, Simmonics, University of Warwick, Coventry, UK) [26,27]. Changes in the tree position of taxa supported at the 70% or greater bootstrap level for a 250 bp sequence window were examined at 100 bp intervals. In addition, a phylogenetic compatibility matrix was constructed and used to examine the frequency and location of recombinations across the entire genome.

### 4.7. Recombination Site Detection

Potential recombination sites were identified using the RDP4 software [22] and a breakpoint map was constructed. A breakpoint density plot was then created from this map by moving a 200 nt window 1 nt at a time along the length of the map. The number of breakpoints falling within a window was plotted at the central window position. A 99% (upper) and 95% (lower) confidence threshold for globally significant breakpoint clusters (defined as windows with more breakpoint positions than the maximum found in >95% of the 1,000 permuted plots) was calculated. In addition, 99% and 95% confidence intervals were calculated for local breakpoint clusters (defined as windows with more breakpoint positions than the maximum found in >99% of the windows at that location in 1,000 permuted plots).

## Figures and Tables

**Table 1.** Genes and coding regions for eight strains of avian infectious bronchitis virus examined in this study.

**ORF^a^**	**CAV/CAV56b/91**			**DE/DE072/92**			**FL/FL18288/71**			**Gray/Gray/60**			**Mass/H120**			**Holte/Holte/54**			**Iowa/Iowa97/56**			**JMK/JMK/64**		
	Location	nt^b^	aa^c^	Location	nt	aa	Location	nt	aa	Location	nt	aa	Location	nt	aa	Location	nt	aa	Location	nt	aa	Location	nt	aa
**5′UTR**	1–527	527	–	1–528	528	–	1–528	528	–	1–528	528	–	1–528	528	–	1–528	528	–	1–528	528	–	1–528	528	–
**1a**	528–12389	1,862	3953	529–12309	11781	3926	529–12387	11859	3952	529–12387	11859	3952	529–12330	11802	3933	529–12384	11856	3951	529–12390	11802	3933	529–12387	11859	3952
**1ab**	528–20422	19895	6631	529–20336	19808	6602	529–20420	19892	6630	529–20420	19892	6630	529–20363	19835	6611	529–20414	19886	6628	529–20423	19895	6631	529–20421	19893	6630
**Spike**	20373–23873	3501	1166	20287–23739	3453	1150	20371–23838	3468	1155	20371–23874	3504	1167	20314–23802	3489	1162	20365–23871	3507	1168	20374–23880	3507	1168	20371–23877	3507	1168
**3a**	23873–24046	174	57	23785–23958	174	57	23838–24011	164	54	23874–24047	174	57	23802–23975	174	57	23871–24044	174	57	23880–24053	174	57	23877–24050	174	57
**3b**	24046–24240	195	64	23958–24152	195	64	24011–24202	192	63	24047–24241	195	64	23975–24169	195	64	24044–24238	195	64	24053–24247	195	64	24050–24244	195	64
**Envelope**	24221–24502	282	93	24133–24462	330	109	24186–24488	303	100	24222–24545	324	107	24150–24479	330	109	24219–24542	324	107	24228–24551	324	107	24225–24548	324	107
**Membrane**	24651–25175	525	174	24434–25111	678	225	24488–25156	669	222	24523–25188	666	221	24451–25128	678	225	24520–25188	667	222	24529–25140	612	203	24526–25197	672	223
**4b**	25176–25460	285	94	25112–25396	285	94	25157–25441	285	94	25189–25428	240	79	25129–25371	243	80	25189–25473	285	94	25194–25478	285	94	25198–25329	132	43
**4c**	25381–25554	174	57	25317–25487	171	56	25362–25532	171	56	25340–25510	171	56	25334–25504	171	56	25394–25534	141	46	25399–25539	141	46	25374–25568	195	64
**5a**	25538–25735	198	65	25471–25668	198	65	25516–25713	198	65	25494–25691	198	65	25488–25685	198	65	25547–25744	198	65	25552–25749	198	65	25552–25749	198	65
**5b**	25732–25980	249	82	25665–25913	249	82	25710–25958	249	82	25688–25936	249	82	25682–25930	249	82	25741–25989	249	82	25746–25994	249	82	25746–25994	249	82
**Nucleocapsid**	25923–27152	1230	409	25856–27085	1230	409	25901–27130	1230	409	25879–27111	1233	410	25873–27102	1230	409	25932–27161	1230	409	25937–27166	1230	409	25937–27166	1230	409
**6b**	27161–27385	225	74	27094–27318	225	74	27139–27363	225	74	–	–	–	27126–27356	231	76	–	–	–	27175–27399	225	74	27175–27399	225	74
**3′UTR**	27386–27663	248	–	27319–27591	273	–	27364–27616	253	–	27112–27568	455	–	27357–27632	276	–	27162–27246	85	–	27340–27662	323	–	27400–27793	393	–

a ORF = open reading frame;

b nt = nucleotide;

c aa = amino acid.

**Table 2.** Recombination breakpoints ^a^, genes and major and minor related sequences in other infectious bronchitis virus (IBV) strains.

**Recombinant**	**Breakpoints**		**Genes^b^**	**Major Sequence^c^**	**Minor Sequence^d^**	**Detection Method**
	**Begin**	**End**				
Ark/Ark-DPI-11/81	3,498	8,667	1ab (nsp 3, 4, and 5)	Conn/Conn46/66	DE/DE072/92	RDP, GENECONV, Maxchi, Chimaera, SiSscan, 3Seq
	4,312	10,590	1ab (nsp 3, 4, 5, and 6)	CK/CH/LSD/051	DE/DE072/92	RDP, GENECONV, Maxchi, Chimaera, SiSscan, 3Seq
	13,072	20,186	1ab (nsp 11/12, 13, 14, 15, and 16)	Unknown^e^ (JMK/JMK/64)	CAL/CAL99/99	RDP, GENECONV, Maxchi, Chimaera, SiSscan, 3Seq
	20,292	23,909	1ab (nsp16), Spike, 3a	Conn/Conn46/66	Unknown (Mass/Mass41)	RDP, GENECONV, Maxchi, Chimaera, SiSscan, 3Seq
	21,613	23,856*	Spike, 3a	CAL/CAL99/99	JMK/JMK/64	RDP, Maxchi, Chimaera, SiSscan, 3Seq
Ark/Ark-DPI-101/81	3,498	8,667	1ab (nsp 3, 4, and 5)	Conn/Conn46/66	DE/DE072/92	RDP, GENECONV, Maxchi, Chimaera, SiSscan, 3Seq
	4,312	10,590	1ab (nsp 3, 4, 5, and 6)	CK/CH/LSD/051	DE/DE072/92	RDP, GENECONV, Maxchi, Chimaera, SiSscan, 3Seq
	13,072	20,186	1ab (nsp 11/12, 13, 14, 15, and 16)	Unknown (JMK/JMK/64)	CAL/CAL99/99	RDP, GENECONV, Maxchi, Chimaera, SiSscan, 3Seq
	20,292	23,909	1ab (nsp16), Spike, 3a	Conn/Conn46/66	Unknown (Mass/Mass41)	RDP, GENECONV, Maxchi, Chimaera, SiSscan, 3Seq
	21,613	23,856*	Spike, 3a	CAL/CAL99/99	JMK/JMK/64	RDP, Maxchi, Chimaera, SiSscan, 3Seq
CAL/CAL99/99	0*	4,368*	5′UTR,1ab (nsp 2 and 3)	Ark/Ark-DPI/81	Unknown (DE/DE072/92)	RDP, GENECONV, Maxchi, Chimaera, SiSscan, 3Seq
	2,382	4,255*	1ab (nsp2,nsp3)	DE/DE072/92	Conn/Conn46/66	RDP, GENECONV, Maxchi, Chimaera, SiSscan, 3Seq
	4,312	10,590	1ab (nsp 3, 4, 5, and 6)	CK/CH/LSD/051	DE/DE072/92	RDP, GENECONV, Maxchi, Chimaera, SiSscan, 3Seq
	8,104	10,649*	1ab (nsp 4, 5, and 6)	DE/DE072/92	Conn/Conn46/66	RDP, Maxchi, Chimaera, SiSscan, 3Seq
	24,587*	25,773	Envelope, Membrane, 4b, 4c, 5a, 5b	Unknown (GA/GA98/98)	Ark/Ark-DPI/81	RDP, GENECONV, Maxchi, Chimaera, SiSscan, 3Seq
CAV/56b/91	0*	1,512	1ab (nsp 2)	ITA/90254/2005	DE/DE072/92	RDP, GENECONV, Maxchi, Chimaera, 3Seq
	0*	4,368*	5′UTR,1ab (nsp 2 and 3)	Ark/Ark-DPI/81	Unknown (DE/DE072/92)	RDP, GENECONV, Maxchi, Chimaera, SiSscan, 3Seq
	4,312	10,590	1ab (nsp 3, 4, 5, and 6)	CK/CH/LSD/051	DE/DE072/92	RDP, GENECONV, Maxchi, Chimaera, SiSscan, 3Seq
	4,392*	4,558	1ab (nsp3)	Ark/Ark-DPI/81	Conn/Conn46/91	GENECONV, Maxchi, Chimaera, 3Seq
	8,104	10,649*	1ab (nsp 4, 5, and 6)	DE/DE072/92	Conn/Conn46/66	RDP, Maxchi, Chimaera, SiSscan, 3Seq
	13,072	20,186	1ab (nsp 11/12, 13, 14, 15, and 16)	Unknown (JMK/JMK/64)	CAL/CAL99/99	RDP, GENECONV, Maxchi, Chimaera, SiSscan, 3Seq
	20,292	23,909	1ab (nsp16), Spike, 3a	Conn/Conn46/66	Unknown (Mass/Mass41)	RDP, GENECONV, Maxchi, Chimaera, SiSscan, 3Seq
	24,556	25,748	Envelope, Membrane, 4b, 4c, 5a, 5b	Ark/Ark-DPI/81	Unknown (CAL/CAL99/99)	RDP, GENECONV, Maxchi, Chimaera, SiSscan, 3Seq
CK/CH/BJ/97	31*	5,600	5′UTR, 1ab (nsp 2 and 3)	CK/CH/SAIBK	Unknown (CK/CH/CQ041/04)	RDP, GENECONV, Maxchi, Chimaera, SiSscan, 3Seq
CK/CH/CQ04-1/04	60*	4,711	5′UTR, 1ab (nsp 2 and 3)	CK/CH/SC021202/02	CK/CH/DY-07/07	RDP, GENECONV, Maxchi, Chimaera, SiSscan, 3Seq
	8,751	9,018	1 ab (nsp 5)	CK/CH/SC021202/02	CK/CH/DY-07/07	RDP, GENECONV, Maxchi, Chimaera
	9,626	18,737	1ab (nsp 5, 6, 7, 8, 9, 10, 11/12, 13, 14, 15)	CK/CH/SAIBK	CK/CH/DY-07/07	RDP, GENECONV, Maxchi, Chimaera, SiSscan, 3Seq
	18,738*	20,350	1ab (nsp 15 and 16)	CK/CH/SAIBK	ITA/90254/2005	RDP, GENECONV, Maxchi, Chimaera
	20,160	21,138	1ab (nsp 16), Spike	JMK/JMK/64	CK/CH/BJ/97	RDP, GENECONV, Maxchi, Chimaera, SiSscan
	27,120	27,354	Nucleocapsid, 6b	JMK/JMK/64	CK/CH/DY-07/07	GENECONV, Maxchi, Chimaera, SiSscan
CK/CH/DY-07/07	1,170	5,017	1ab (nsp 2 and 3)	DE/DE072/92	CK/CH/SAIBK	RDP, GENECONV, Maxchi, Chimaera, SiSscan, 3Seq
	22,216	23,963	Spike, 3a	CK/CH/BJ/97	CK/CH/CQ04-1/04	RDP, GENECONV, Maxchi, Chimaera, SiSscan, 3Seq
	25,455	25,662	4c, 5a	CK/CH/BJ/97	CK/CH/CQ04-1/04	RDP, GENECONV, Maxchi, Chimaera, SiSscan
CK/CH/LSD/051/06	306	3,628*	5′UTR, 1ab (nsp 2 and 3)	Mass/Mass41	Ark/Ark-DPI/81	RDP, GENECONV, Maxchi, Chimaera, SiSscan, 3Seq
	1,453	2,743	1ab (nsp 2 and 3)	Mass/H52	Mass/Mass41/41	GENECONV, Maxchi, Chimaera, 3Seq
	13,668	14,734	1ab, (nsp 11/12)	Mass/Mass41/41	DE/DE072/92	RDP, GENECONV, Maxchi, Chimaera, SiSscan, 3Seq
	15,447	15,821	1ab (nsp 13)	Mass/Mass41/41	DE/DE072/92	RDP, GENECONV, Maxchi, Chimaera, SiSscan
	20,203	24,772	1ab (nsp 16), Spike, 3a, 3b, Envelope, Membrane	NGA/A116E7/06	Mass/Mass41	RDP, GENECONV, Maxchi, Chimaera, SiSscan, 3Seq
	25,063	25,776	Membrane, 4b, 4c, 5a, 5b	Unknown (Mass/Mass41/41)	Mass/H120	RDP, GENECONV, Maxchi, Chimaera, SiSscan, 3Seq
	25,774*	26,341	5b, Nucleocapsid	Mass/Mass41/41	Mass/H120	RDP, GENECONV, SiSscan, 3Seq
CK/CH/SAIBK	7,241	9,126	1ab (nsp 3, 4,5)	CK/CH/SC0212/02	DE/DE072/92	RDP, GENECONV, Maxchi, Chimaera, SiSscan, 3Seq
	20,160	21,138	1ab (nsp 16), Spike	JMK/JMK/64	CK/CH/BJ/97	RDP, GENECONV, Maxchi, Chimaera, SiSscan
CK/CH/SC021202/02	13,342	14,784	1ab (nsp 11/12)	CK/CH/SAIBK	CK/CH/DY-07/07	RDP, GENECONV, Maxchi, Chimaera, SiSscan, 3Seq
	20,160	21,138	1ab (nsp 16), Spike	JMK/JMK/64	CK/CH/BJ/97	RDP, GENECONV, Maxchi, Chimaera, SiSscan
	27,120	27,354	Nucleocapsid, 6b	JMK/JMK/64	CK/CH/DY-07/07	GENECONV, Maxchi, Chimaera, SiSscan
CK/CH/ZJ971/97	0*	11,115	5′UTR, 1ab (nsp 2, 3, 4, 5, 6, 7, and 8)	NGA/A116E7/06	Ark/Ark-DPI/81	RDP, GENECONV, Maxchi, Chimaera, SiSscan
	306	3,628*	5′UTR, 1ab (nsp 2 and 3)	Mass/Mass41	Ark/Ark-DPI/81	RDP, GENECONV, Maxchi, Chimaera, SiSscan, 3Seq
	4,312	10,590	1ab (nsp 3, 4, 5, and 6)	CK/CH/LSD/051	DE/DE072/92	RDP, GENECONV, Maxchi, Chimaera, SiSscan, 3Seq
	20,203	24,772	1ab (nsp 16), Spike, 3a, 3b, Envelope, Membrane	NGA/A116E7/06	Mass/Mass41	RDP, GENECONV, Maxchi, Chimaera, SiSscan, 3Seq
	26,286	27,027	Nucleocapsid, 6b, 3′UTR	Iowa/Iowa97/56	CAL/CAL99/99	RDP, GENECONV, Maxchi, Chimaera, 3Seq
	27,094	27,244	Nucleocapsid, 6b	Iowa/Iowa97/56	Unknown (TW/2575/98)	RDP, GENECONV, Maxchi, Chimaera, SiSscan
Conn/Conn46/66	0*	1,512	1ab (nsp 2)	ITA/90254/2005	DE/DE072/92	RDP, GENECONV, Maxchi, Chimaera, 3Seq
	0*	4,368*	5′UTR,1ab (nsp 2 and 3)	Ark/Ark-DPI/81	Unknown (DE/DE072/92)	RDP, GENECONV, Maxchi, Chimaera, SiSscan, 3Seq
	13,072	20,186	1ab (nsp 11/12, 13, 14, 15, and 16)	Unknown (JMK/JMK/64)	CAL/CAL99/99	RDP, GENECONV, Maxchi, Chimaera, SiSscan, 3Seq
	20,361	21,981	Spike	CAL/CAL99/99	Mass/Mass41	RDP, GENECONV, Maxchi, Chimaera, SiSscan, 3Seq
Conn/Conn46/91	0*	1,512	1ab (nsp 2)	ITA/90254/2005	DE/DE072/92	RDP, GENECONV, Maxchi, Chimaera, 3Seq
	0*	4,368*	5′UTR,1ab (nsp 2 and 3)	Ark/Ark-DPI/81	Unknown (DE/DE072/92)	RDP, GENECONV, Maxchi, Chimaera, SiSscan, 3Seq
	13,072	20,186	1ab (nsp 11/12, 13, 14, 15, and 16)	Unknown (JMK/JMK/64)	CAL/CAL99/99	RDP, GENECONV, Maxchi, Chimaera, SiSscan, 3Seq
	20,361	21,981	Spike	CAL/CAL99/99	Mass/Mass41	RDP, GENECONV, Maxchi, Chimaera, SiSscan, 3Seq
DE/DE072/92	0*	11,115	5′UTR, 1ab (nsp 2, 3, 4, 5, 6, 7, and 8)	NGA/A116E7/06	Ark/Ark-DPI/81	RDP, GENECONV, Maxchi, Chimaera, SiSscan
	18,776	19,911*	1ab (nsp 15 and 16)	Mass/H120	Ark/Ark-DPI/81	RDP, GENECONV, Maxchi, Chimaera, SiSscan, 3Seq
	19,934	24,431	1ab (nsp16), Spike, 3a, 3b, Envelope	Mass/H120	Unknown (Mass/Mass41)	RDP, GENECOV, Maxchi, Chimaera, SiSscan, 3Seq
	20,203	24,772	1ab (nsp 16), Spike, 3a, 3b, Envelope, Membrane	NGA/A116E7/06	Mass/Mass41	RDP, GENECONV, Maxchi, Chimaera, SiSscan, 3Seq
	23,504	24,431*	Spike, 3a, 3b, Envelope	CK/CH/CQ04-1/04	CALCAL99/99	RDP, GENECONV, Maxchi, Chimaera, SiSscan, 3Seq
	25,575	27,482*	5a, 5b, Nucleocapsid, 6b, 3′UTR	CK/CH/ZJ971/97	JMK/JMK/64	RDP, GENECONV, Maxchi, Chimaera, SiSscan, 3Seq
FL/FL18288/71	0*	1,512	1ab (nsp 2)	ITA/90254/2005	DE/DE072/92	RDP, GENECONV, Maxchi, Chimaera, 3Seq
	0*	4,368*	5′UTR,1ab (nsp 2 and 3)	Ark/Ark-DPI/81	Unknown (DE/DE072/92)	RDP, GENECONV, Maxchi, Chimaera, SiSscan, 3Seq
	13,072	20,186	1ab (nsp 11/12, 13, 14, 15, and 16)	Unknown (JMK/JMK/64)	CAL/CAL99/99	RDP, GENECONV, Maxchi, Chimaera, SiSscan, 3Seq
	20,361	21,981	Spike	CAL/CAL99/99	Mass/Mass41	RDP, GENECONV, Maxchi, Chimaera, SiSscan, 3Seq
GA98/0470/98	0*	4,368*	5′UTR,1ab (nsp 2 and 3)	Ark/Ark-DPI/81	Unknown (DE/DE072/92)	RDP, GENECONV, Maxchi, Chimaera, SiSscan, 3Seq
	2,382	4,255*	1ab, (nsp2 and 3)	DE/DE072/92	Conn/Conn46/66	RDP, GENECONV, Maxchi, Chimaera, SiSscan, 3Seq
	3,498	8,667	1ab (nsp 3, 4, and 5)	Conn/Conn46/66	DE/DE072/92	RDP, GENECONV, Maxchi, Chimaera, SiSscan, 3Seq
	9,569	9,770	1ab (nsp 5)	Gray/Gray/60	Unknown (NGA/A116E7/06)	RDP, GENECONV, Maxchi, Chimaera, SiSscan, 3Seq
	13,072	20,186	1ab (nsp 11/12, 13, 14, 15, and 16)	Unknown (JMK/JMK/64)	CAL/CAL99/99	RDP, GENECONV, Maxchi, Chimaera, SiSscan, 3Seq
	23,504	24,431*	Spike, 3a, 3b, Envelope	CK/CH/CQ04-1/04	CALCAL99/99	RDP, GENECONV, Maxchi, Chimaera, SiSscan, 3Seq
	24,500*	25,438	Membrane, 4b	Conn/Conn46/66	Mass/Mass41/41	RDP, GENECONV, Chimaera, SiSscan, 3Seq
Gray/Gray/60	0*	4,368*	5′UTR,1ab (nsp 2 and 3)	Ark/Ark-DPI/81	Unknown (DE/DE072/92)	RDP, GENECONV, Maxchi, Chimaera, SiSscan, 3Seq
	8,488	12,055	1ab (nsp 4, 5, 6, 7, 8, 9, and 10)	Unknown (CK/CH/LSD/051/06)	Conn/Conn46/91	RDP, GENECONV, Maxchi, Chimaera
	13,070*	14,216	1ab (nsp 11/12)	Unknown (CAV/56b/91)	Ark/Ark-DPI/81	RDP, GENECONV, Maxchi, Chimaera, SiSscan, 3Seq
	24,131	27,145	3b, Envelope, Membrane, 4b, 4c, 5a, 5b, Nucleocapsid, 3′UTR	Ark/Ark-DPI/81	Unknown (Conn/Conn46/91)	RDP, GENECONV, Maxchi, Chimaera, SiSscan, 3Seq
Holte/Holte/54	0*	4,368*	5′UTR,1ab (nsp 2 and 3)	Ark/Ark-DPI/81	Unknown (DE/DE072/92)	RDP, GENECONV, Maxchi, Chimaera, SiSscan, 3Seq
Iowa/Iowa97/56	0*	4,368	5′UTR,1ab (nsp 2 and 3)	Ark/Ark-DPI/81	Unknown (DE/DE072/92)	RDP, GENECONV, Maxchi, Chimaera, SiSscan, 3Seq
	4,368	5,144	1ab (nsp 3)	Holte/Holte/54	DE/DE072/92	RDP, GENECONV, Maxchi, Chimaera, SiSscan, 3Seq
ITA/90254/05	16,367	25,699	1ab (nsp 13, 14, 15, 16) Spike, 3a, 3b, Envelope, Membrane, 4b, 4c, 5a	GA98/0470/98	CK/CH/BJ/97	RDP, GENCOV, Maxchi, SiSscan
	22,216	23,963	Spike, 3a	CK/CH/BJ/97	CK/CH/CQ04-1/04	RDP, GENECONV, Maxchi, Chimaera, SiSscan, 3Seq
	24,423	25,632*	Envelope, Membrane, 4b, 4c, 5a	CK/CH/DY-07/07	NGA/A116E7/06	RDP, GENECONV, Maxchi, Chimaera, 3Seq
JMK/JMK/64	0*	1,512	1ab (nsp 2)	ITA/90254/2005	DE/DE072/92	RDP, GENECONV, Maxchi, Chimaera, 3Seq
	0*	4,368*	5′UTR,1ab (nsp 2 and 3)	Ark/Ark-DPI/81	Unknown (DE/DE072/92)	RDP, GENECONV, Maxchi, Chimaera, SiSscan, 3Seq
	8,488	12,055	1ab (nsp 4, 5, 6, 7, 8, 9, and 10)	Unknown (CK/CH/LSD/051/06)	Conn/Conn46/91	RDP, GENECONV, Maxchi, Chimaera
	13,070*	14,216	1ab (nsp 11/12)	Unknown (CAV/56b/91)	Ark/Ark-DPI/81	RDP, GENECONV, Maxchi, Chimaera, SiSscan, 3Seq
	24,131	27,145	3b, Envelope, Membrane, 4b, 4c, 5a, 5b, Nucleocapsid, 3′UTR	Ark/Ark-DPI/81	Unknown (Conn/Conn46/91)	RDP, GENECONV, Maxchi, Chimaera, SiSscan, 3Seq
Mass/H52	306	3,628*	5′UTR, 1ab (nsp 2 and 3)	Mass/Mass41	Ark/Ark-DPI/81	RDP, GENECONV, Maxchi, Chimaera, SiSscan, 3Seq
	4,312	10,590	1ab (nsp 3, 4, 5, and 6)	CK/CH/LSD/051	DE/DE072/92	RDP, GENECONV, Maxchi, Chimaera, SiSscan, 3Seq
	19,925	20,168*	1ab (nsp 16)	Mass/Mass41/41	Mass/H120	GENECONV, Maxchi, Chimaera, SiSscan
	20,203	24,772	1ab (nsp 16), Spike, 3a, 3b, Envelope, Membrane	NGA/A116E7/06	Mass/Mass41/41	RDP, GENECONV, Maxchi, Chimaera, SiSscan, 3Seq
	25,063	25,776	Membrane, 4b, 4c, 5a, 5b	Unknown (Mass/Mass41/41)	Mass/H120	RDP, GENECONV, Maxchi, Chimaera, SiSscan, 3Seq
	26,286	27,027	Nucleocapsid, 6b, 3′UTR	Iowa/Iowa97/56	CAL/CAL99/99	RDP, GENECONV, Maxchi, Chimaera, 3Seq
	26,372	27,526*	Nucleocapsid, 6b, 3′UTR	Unknown (DE/DE072/92)	Mass/H120	RDP, GENECONV, Maxchi, Chimaera, SiSscan, 3Seq
	27,094	27,244	Nucleocapsid, 6b	Iowa/Iowa97/56	Unknown (TW/2575/98)	RDP, GENECONV, Maxchi, Chimaera, SiSscan
Mass/H120	0*	11,115	5′UTR, 1ab (nsp 2, 3, 4, 5, 6, 7, and 8)	NGA/A116E7/06	Ark/Ark-DPI/81	RDP, GENECONV, Maxchi, Chimaera, SiSscan
	306	3,628*	5′UTR, 1ab (nsp 2 and 3)	Mass/Mass41	Ark/Ark-DPI/81	RDP, GENECONV, Maxchi, Chimaera, SiSscan, 3Seq
	4,312	10,590	1ab (nsp 3, 4, 5, and 6)	CK/CH/LSD/051	DE/DE072/92	RDP, GENECONV, Maxchi, Chimaera, SiSscan, 3Seq
	20,203	24,772	1ab (nsp 16), Spike, 3a, 3b, Envelope, Membrane	NGA/A116E7/06	Mass/Mass41	RDP, GENECONV, Maxchi, Chimaera, SiSscan, 3Seq
	26,286	27,027	Nucleocapsid, 6b, 3′UTR	Iowa/Iowa97/56	CAL/CAL99/99	RDP, GENECONV, Maxchi, Chimaera, 3Seq
	27,094	27,244	Nucleocapsid, 6b	Iowa/Iowa97/56	Unknown (TW/2575/98)	RDP, GENECONV, Maxchi, Chimaera, SiSscan
NGA/A116E7/06	7,035	8,271	1ab (nsp 3 and 4)	Holte/Holte/54	DE/DE072/92	RDP, GENECONV, Maxchi, Chimaera, 3Seq
TW/2575/98	20,160	21,138	1ab (nsp 16), Spike	JMK/JMK/64	CK/CH/BJ/97	RDP, GENECONV, Maxchi, Chimaera, SiSscan
	27,120	27,354	Nucleocapsid, 6b	JMK/JMK/64	CK/CH/DY-07/07	GENECONV, Maxchi, Chimaera, SiSscan

* The actual breakpoint position is undetermined. Most likely it was overprinted by a subsequent recombination event.

a Only transferred gene fragments with statistical support of >1 × 10^−12^ (50 of 135 total unique fragments) are included in the table.

b Genes indicate the coding sequences contained within the fragment introduced by recombination.

c Major Sequence = Sequence most closely related to the sequence surrounding the transferred fragment.

d Minor Sequence = Sequence closely related to the transferred fragment in the recombinant.

e Unknown = only one parent and a recombinant need be in the alignment for a transferred fragment to be detectable. The sequence listed in parentheses was used to infer the existence of a missing parental sequence.

**Table 3.** Number of transferred fragments associated with individual areas of the genome for all of the strains examined.

**Genomic Region**	**Number of Fragments^a^**	**% of Total**
5′UTR ^b^	8	4.2
Nsp ^c^ 2	20	10.5
nsp 3	33	17.3
nsp 4	17	8.9
nsp 5	15	7.9
nsp6	10	8.3
nsp 7	6	3.2
nsp 8	6	3.2
nsp9	4	2.1
nsp 10	4	2.1
nsp 11/12	13	6.8
nsp 13	12	6.3
nsp 14	10	5.3
nsp 15	10	5.3
nsp 16	19	10.0
Spike	30	15.8
3a	14	7.4
3b	13	6.8
Envelope	17 8.9	
Membrane	17	8.9
4b	12	6.3
4c	12	6.3
5a	15	7.9
5b	11	5.8
Nucleocapsid	14	7.4
3′UTR	13	6.8

a Genomic areas may be fully or only partially located in the transferred fragments.

b UTR = untranslated region.

c nsp = nonstructural protein.

**Table 4.** Viruses sequenced in this study.

**Strain**	**Serotype**	**Origin**	**Source**
CAV/CAV56b/91	CAV	California, USA	P. Woolcock ^a^
DE/DE072/92	DE	Delmarva, USA	J. Gelb Jr ^b^
FL/FL18288/71	FL	Florida, USA	P. Villegas ^c^
Gray/Gray/60	Gray	Delmarva, USA	J. Gelb Jr.
Holte/Holte/54	Holte	Wisconsin, USA	J. King ^d^
Iowa/Iowa97/56	Iowa	Iowa, USA	J. King
JMK/JMK/64	JMK	Delmarva, USA	J. Gelb, Jr.
Mass/H120	Mass	The Netherlands	J. King

a University of California, Davis, CA, USA.

b University of Delaware, Newark, DE, USA.

c University of Georgia, Athens, GA, USA.

d Southeast Poultry Research Laboratory, USDA/ARS, Athens, GA, USA.

## References

[1] Lai MMC, Holmes KV, Knipe DM, Howley PM, Griffin DE, Lamb RA, Martin MA, Roizman B, Straus SE (2001). Coronaviridae: The viruses and their replication. Fields Virology.

[2] Cavanagh D, Mawditt K, Adzhar A, Gough RE, Picault JP, Naylor CJ, Haydon D, Shaw K, Britton P (1998). Does IBV change slowly despite the capacity of the spike protein to vary greatly. Adv Exp Med Biol.

[3] Niesters HG, Kusters JG, Lenstra JA, Spaan WJ, Horzined MC, van der Zeijst BA (1987). The neutralization epitopes on the spike protein of infectious bronchitis virus and their antigenic variation. Adv Exp Med Biol.

[4] Holmes EC (2009). The Evolution and Emergence of RNA Viruses.

[5] Lai M (1992). RNA Recombination in animal and plant viruses. Microbiol Rev.

[6] Kusters JG, Jager EJ, Niesters HGM, van der Zeijst BAM (1990). Sequence evidence for RNA recombination in field isolates of avian coronavirus infectious bronchitis virus. Vaccine.

[7] Jia W, Karaca K, Parrish CR, Naqi SA (1995). A novel variant of avian infectious bronchitis virus resulting from recombination among three different strains. Arch Virol.

[8] Lee CW, Jackwood MW (2001). Origin and evolution of Georgia 98 (GA98), a new serotype of avian infectious bronchitis virus. Virus Res.

[9] Lee CW, Jackwood MW (2000). Evidence of genetic diversity generated by recombination among avian coronavirus IBV. Arch Virol.

[10] Estevez C, Villegas P, El-Attrache J (2003). A recombination event, induced in ovo, between a low passage infectious bronchitis virus field isolate and a highly embryo adaptedvaccine strain. Avian Dis.

[11] Mardani K, Noormohammadi AH, Ignjatovic J, Browning GF (2010). Naturally occurring recombination between distant strains of infectious bronchitis virus. Arch Virol.

[12] Woo PC, Lau SK, Huang Y, Yuen KY (2009). Coronavirus diversity, phylogeny and interspecies jumping. Exp Biol Med.

[13] Decaro N, Mari V, Campolo M, Lorusso A, Camero M, Elia G, Martella V, Cordioli P, Enjuanes L, Buonavoglia C (2009). Recombinant canine coronaviruses related to transmissible gastroenteritis virus of Swine are circulating in dogs. J Virol.

[14] Jackwood MW, Boynton TO, Hilt DA, McKinley ET, Kissinger JC, Paterson AH, Robertson J, Lemke C, McCall AW, Williams SM, Jackwood JW, Byrd LA (2010). Emergence of a group 3 coronavirus through recombination. Virology.

[15] Lee CW, Hilt DA, Jackwood MW (2001). Identification and analysis of the Georgia 98 serotype, a new serotype of infectious bronchitis virus. Avian Dis.

[16] National Center for Biotechnology Information http://www.ncbi.nlm.nih.gov/.

[17] Tamura K, Dudley J, Nei M, Kumar S (2007). MEGA4: Molecular evolutionary genetics analysis (MEGA) software version 4.0. Mol Biol Evol.

[18] Woo PCY, Huang Y, Lau SK, Yuen KY (2010). Coronavirus genomics and bioinformatics analysis. Viruses.

[19] Worobey M, Holmes EC (1999). Evolutionary aspects of recombination in RNA viruses. J Gen Virol.

[20] Bruen TC, Philippe H, Bryant D (2006). A simple and robust statistical test for detecting the presence of recombination. Genetics.

[21] Heath L, van der Walt E, Varsani A, Martin DP (2006). Recombination patterns in aphthoviruses mirror those found in other picornaviruses. J Virol.

[22] Martin DP (2009). Recombination detection and analysis using RDP3. Methods Mol Biol.

[23] Zhang Y, Wang H-N, Wang T, Fan W-Q, Zhang A-Y, Wei K, Tian G-B, Yang X (2010). Complete genome sequence and recombination analysis of infectious bronchitis virus attenuated vaccine strain H120. Virus Genes.

[24] Hein R (2010).

[25] Woo PCY, Lau SKP, Yip CCY, Huang Y, Tsoi HW, Yuen KY (2006). Comparative analysis of 22 coronavirus HKU1 genomes reveals a novel genotype and evidence of natural recombination in coronavirus HKU1. J Virol.

[26] Simmonds P, Welch J (2006). Frequency and dynamics of recombination within different species of human enteroviruses. J Virol.

[27] Simmonds P, Midgley S (2005). Recombination in the genesis and evolution of hepatitis B virus genotypes. J Virol.

[28] Armesto M, Cavanagh D, Britton P (2009). The replicase gene of avian coronavirus infectious bronchitis virus is a determinant of pathogenicity. PLoS One.

[29] Hagemeijer MC, Verheije MH, Ulasli M, Shaltiel IA, de Vries LA, Reggiori F, Rottier PJ, de Haan CA (2010). Dynamics of coronavirus replication-transcription complexes. J Virol.

[30] Lindner HA, Fotouhi-Ardakani N, Lytvyn V, Lachance P, Sulea T, Menard R (2005). The papain-like protease from the severe acute respiratory syndrome coronavirus is a deubiquitinating enzyme. J Virol.

[31] Zheng D, Chen G, Guo B, Cheng G, Tang H (2008). PLP2, a potent deubiquitinase from murine hepatitis virus, strongly inhibits cellular type I interferon production. Cell Res.

[32] Gorbalenya AE, Koonin EV, Donchenko AP, Blinov VM (1989). Coronavirus genome: Prediction of putative functional domains in the non-structural polyprotein by comparative amino acid sequence analysis. Nucleic Acids Res.

[33] Gorbalenya AE, Koonin EV, Lai MM (1991). Putative papain-related thiol proteases of positive-strand RNA viruses. Identification of rubi- and aphthovirus proteases and delineation of a novel conserved domain associated with proteases of rubi-, alpha- and coronaviruses. FEBS Lett.

[34] Eriksson KK, Cervantes-Barragan L, Ludewig B, Thiel V (2008). Mouse hepatitis virus liver pathology is dependent on ADP-ribose-1″-phosphatase, a viral function conserved in the alpha-like supergroup. J Virol.

[35] Zhang XW, Yap YL, Danchin A (2005). Testing the hypothesis of a recombinant origin of the SARS-associated coronavirus. Arch Virol.

[36] Chen X, Zhang Q, Li J, Cao W, Zhang JX, Zhang L, Zhang W, Shao ZJ, Yan Y (2010). Analysis of recombination and natural selection in human enterovirus 71. Virology.

[37] Gelb JJ, Jackwood MW, Dufour-Zavala L, Swayne DE, Glisson JR, Pearson JE, Reed WM, Jackwood MW, Woolcock P (2008). Infectious Bronchitis. A Laboratory Manual for the Isolation, Identification, and Characterization of Avian Pathogens.

[38] Huson DH, Bryant D (2006). Application of phylogenetic networks in evolutionary studies. Mol Biol Evol.

[39] Bryant D, Moulton V (2004). Neighbor-net: An agglomerative method for the construction of phylogenetic networks. Mol Biol Evol.

[40] Martin DP, Posada D, Crandall KA, Williamson C (2005). A modified bootscan algorithm for automated identification of recombinant sequences and recombination breakpoints. AIDS Res Hum Retroviruses.

[41] Padidam M, Sawyer S, Fauquet CM (1999). Possible emergence of new geminiviruses by frequent recombination. Virology.

[42] Smith JM (1992). Analyzing the mosaic structure of genes. J Mol Evol.

[43] Posada D, Crandall KA (2001). Evaluation of methods for detecting recombination from DNA sequences: Computer simulations. Proc Natl Acad Sci U S A.

[44] Gibbs MJ, Armstrong JS, Gibbs AJ (2000). Sister-scanning: A Monte Carlo procedure for assessing signals in recombinant sequences. Bioinformatics.

[45] Boni MF, Posada D, Feldman MW (2007). An exact nonparametric method for inferring mosaic structure in sequence triplets. Genetics.

[46] Posada D (2002). Evaluation of methods for detecting recombination from DNA sequences: Empirical data. Mol Biol Evol.

[47] Salminen MAM, Lemey P, Salemi M, Vandamme AM (2010). Detecting and characterising individual recombination events: practice. The Phylogenetic Handbook: A Practical Approach to Phylogenetic Analysis and Hypothesis Testing.

